# Association between walking and square dancing-oriented leisure-time physical activity and cognitive function among middle-aged and elderly people in Southwest China

**DOI:** 10.1186/s12877-023-03737-0

**Published:** 2023-01-16

**Authors:** Xu Li, Anjiao Peng, Lin Li, Lei Chen

**Affiliations:** 1 Department of Clinical Research and Management, Center of Biostatistics, Design, Measurement and Evaluation (CBDME), West China Hospital, Sichuan University, Chengdu, Sichuan 610041 People’s Republic of China; 2 Department of Neurology, Joint Research Institute of Altitude Health, West China Hospital, Sichuan University, Chengdu, Sichuan 610041 People’s Republic of China; 3 Med-X Center for Informatics, Sichuan University, Chengdu, Sichuan 610041 People’s Republic of China

**Keywords:** Leisure-time physical activity, Walking, Square dancing, MMSE, Cognitive function

## Abstract

**Background:**

Southwest China is facing a serious aging problem across the country, but the status of cognitive function in middle-aged and elderly people in this region is superior to the national average. This study intends to reveal the leisure-time physical activity (LTPA) pattern in this region and explore whether this pattern is beneficial for cognitive function.

**Methods:**

The data came from the 2019–2021 baseline survey on cognitive function of a natural population cohort conducted by West China Hospital of Sichuan University. A structured questionnaire was used to investigate the LTPA status of the participants, and the Mini-Mental State Examination was used to evaluate their cognitive function. Then, we used multiple linear regression to analyze the association between LTPA and cognitive level, and further subgroup analysis was carried out according to sex, age and waist-to-hip ratio.

**Results:**

A total of 2697 participants were enrolled, with an average age of 66.19 ± 6.68 years. The average cognitive function score was 27.23 ± 2.72, of which 8.60% indicated mild cognitive impairment. Their median LTPA level was 24.50 MET-hours per week, of which 70.37% reached the activity level recommended by WHO, with the main types being walking (1340 cases, 49.68%), square dancing (270 cases, 10.01%), or walking + square dancing (172 cases, 6.38%). Multiple linear regression showed that cognitive function increased with the amount of LTPA from 11.25 MET-hours/week to 36.40 MET-hours/week (*β* 0.09 for 11.25 ~ 24.50 MET-hours/week, *β* 0.38 for 24.50 ~ 36.40 MET-hours/week) but stabilized at more (*β* 0.39 for ≥36.40 MET-hours/week). The positive association persisted even for those who only walked (*β* 0.37 for 24.50 ~ 36.40 MET-hours/week, *P* = 0.008).

**Conclusions:**

Middle-aged and elderly people in Southwest China hold a relatively high level of LTPA status, and walking and square dancing-oriented LTPA are positively correlated with cognitive function.

**Supplementary Information:**

The online version contains supplementary material available at 10.1186/s12877-023-03737-0.

## Background

The world is facing the great challenge of aging. By 2050, there will be 2.1 billion people aged 60 or older worldwide, outnumbering adolescents and young people aged 15–24 [1]. Cognitive disorders, including dementia and mild cognitive impairment (MCI), are one of the health problems associated with aging and have been regarded as a global health priority for the elderly by the WHO [2]. According to the World Alzheimer Report 2021, more than 55 million people worldwide suffer from early/late-onset dementia, and this number will reach 78 million by the year 2030 [3]. In China, dementia and MCI are also highly prevalent, with an approximately 6.0% prevalence for dementia and 15.5% for MCI among people aged over 60 years, representing 15.07 million and 38.77 million people, respectively [4]. However, the prevalence of dementia seems to vary geographically. In the southwestern region, including Sichuan Province, the prevalence of dementia was approximately 4.74% in adults older than 60 years, which is lower than the national average [4].

It was suggested that the regional differences in cognitive dysfunction may be due to distinctions in individual behaviors, life experiences and health conditions [5]. The Lancet Commission estimated that 12 modifiable dementia risk factors, including unhealthy individual behavior, such as insufficient physical activity, could account for approximately 40% of the global risk of dementia [6]. And it was strongly recommended by the WHO that older adults should increase their level of physical activity to reduce the risk of cognitive decline [7], and at least 150 min/week of moderate intensity, 75 min/week of vigorous intensity leisure-time physical activity (LTPA), or an equivalent combination of the two were suggested for potential health benefits [8].

There are many types of LTPA that middle-aged and elderly people prefer, such as aerobic exercise (walking, jogging, dancing, etc.), and balance training exercises (Tai Chi and yoga, etc.) [9]. However, due to regional and cultural differences, LTPA patterns of people in different regions may not be alike. According to the 2018 Sports Preference Map of China’s Provinces, adults in northern regions, such as Beijing and Ningxia Provinces, prefer playing basketball and yoga, while adults in eastern regions, such as Jiangsu and Zhejiang Provinces, prefer running [10]. To date, no relevant studies have reported in detail the real-world LTPA patterns of middle-aged and elderly people over 45 years old in Southwest China.

Sichuan Province of Southwest China has entered a stage of deep aging. According to the “Thirteenth Five-Year Plan” Aging Development Report of the local government [11], Sichuan Province had a population of 18.164 million people aged 60 and above in 2020, ranking third in the country. Considering that the region had a serious aging problem but held a relatively superior status of cognitive function than the national average, we wanted to know the LTPA pattern in middle-aged and elderly people in this region and whether this pattern can improve cognitive function as expected by the WHO.

Therefore, based on a natural population cohort established by West China Hospital, this study is the first to explore the current status of LTPA in middle-aged and elderly people in Southwest China, aiming to comprehensively reveal the characteristics of LTPA in people over 45 years old in this region and its association with cognitive function from the aspects of LTPA type, frequency and amounts, so as to provide a reference for cognitive-related PA intervention among the elderly population.

## Methods

### Study design and data sources

This cross-sectional data stemmed from a Southwest China cohort study conducted by West China Hospital, Sichuan University. The natural population cohort study of West China Hospital used cluster sampling, which considered economic development status and geographical region, in the cities and counties where the medical alliance hospital is located to enroll residents aged 20 years or older in Sichuan Province. Baseline health information was collected through a series of questionnaires, physical examinations, biological sample collection and clinical examinations, and then 10 years of follow-up would be conducted, to establish a multidimensional, dynamic and quantitative health big data platform for health prevention in Southwest China.

To date, the cohort has completed the collection of baseline information in the cities of Chengdu, Mianzhu, and Ganzi from 2019 to 2021. Our cross-sectional study included baseline participants who were 45 years old or older and had completed the assessment for cognitive function. The study was approved by the ethical community of West China Hospital, Sichuan University, and all participants signed informed consent forms.

### Measurement of leisure-time physical activity

Self-reported LTPA was measured using a standard structured questionnaire, with questions including regularly participating in LTPA during the past 6 months or not, type of LTPA (walking, square dancing [12], ball games, etc.), frequency of LTPA, and amount of time spent on LTPA. We used metabolic equivalents (METs) to assess the LTPA level of each activity according to the 2011 Compendium of physical activities [13] (supplementary Table 1), and the product of METs and duration (hours) yields the amount of physical activity. For the total amount of LTPA, we summed the MET-hours per week across all activity types engaged in. For the total duration of LTPA, we summed the hours per week across all activity types.

Light, moderate, and vigorous-intensity activities correspond to 1.1 ~ 2.9 METs, 3.0 ~ 5.9 METs, and ≥ 6 METs, respectively [14]. According to the WHO recommendation, physical activity was defined as at least 150 min/week of moderate-intensity LTPA, 75 min/week of vigorous-intensity LTPA, or an equivalent combination of the two. For convenience of calculation, we assigned moderate intensity LTPA to be 4.5 METs; thus, the recommendation level was at least 11.25 MET-hours/week.

### Assessment of cognitive function

The Mini-Mental State Examination (MMSE) [15, 16] was used to evaluate the cognitive function of participants, which comprised 5 domains, including orientation to time and place (10 points), registration (3 points), attention and calculation (5 points), recall (3 points), and language (9 points). The total score ranged from 0 to 30, and a higher score indicated better cognitive function. An MMSE score < 24 was defined as MCI [17].

### Measurement of covariates

Covariates including age, marital status, gender, education level, smoking status, drinking status, diet, and status of diabetes, depression, anxiety, sleep quality, stroke, cancer, and traumatic brain injuries were collected by self-report questionnaires. Fasting blood was collected for routine biochemical detection to evaluate health status, such as diabetes. The status of hypertension, body mass index (BMI) and waist-to-hip ratio (WHR) were assessed by objective measurements. We used the Patient Health Questionnaire-9 (PHQ-9) to evaluate depression status [18] and the 7-item Generalized Anxiety Disorder scale (GAD-7) to measure anxiety status [19], with each of the total scores ≥5 as the cutoff point for the diagnosis of minor depression or anxiety [20]. When measuring sleep quality, the Pittsburgh Sleep Quality Index (PSQI) was used [21], and participants were graded into three groups according to their PSQI scores: good (0–5), not bad (6–10), and general/poor (11–21). BMI was defined as weight in kilograms divided by height squared in meters, and BMI ≥ 24 was regarded as overweight [22]. WHR was defined as waist circumference divided by hip circumference, and WHR ≥ 0.9 (for men) and ≥ 0.85 (for women) were regarded as central obesity [23].

### Statistical analysis

Descriptive statistics were used to present the characteristics of the participants. Categorical variables were presented as frequencies (composition ratio). Continuous variables were presented as the mean and standard deviation (SD). The correlation between LTPA and cognitive function was examined using linear regression with a robust standard error model. First, we used a univariate model to select variables, with *p* < 0.1 as a selection criterion. Then, we performed multiple analyses to show the relationship between LTPA and cognitive function after adjusting for potentially confounding variables. Further subgroup analyses were conducted according to sex, age and WHR.

All the analyses in this study were carried out by R 4.1.2 software, and *p* < 0.05 was considered statistically significant.

## Results

### Characteristics of participants

**Fig. 1:**
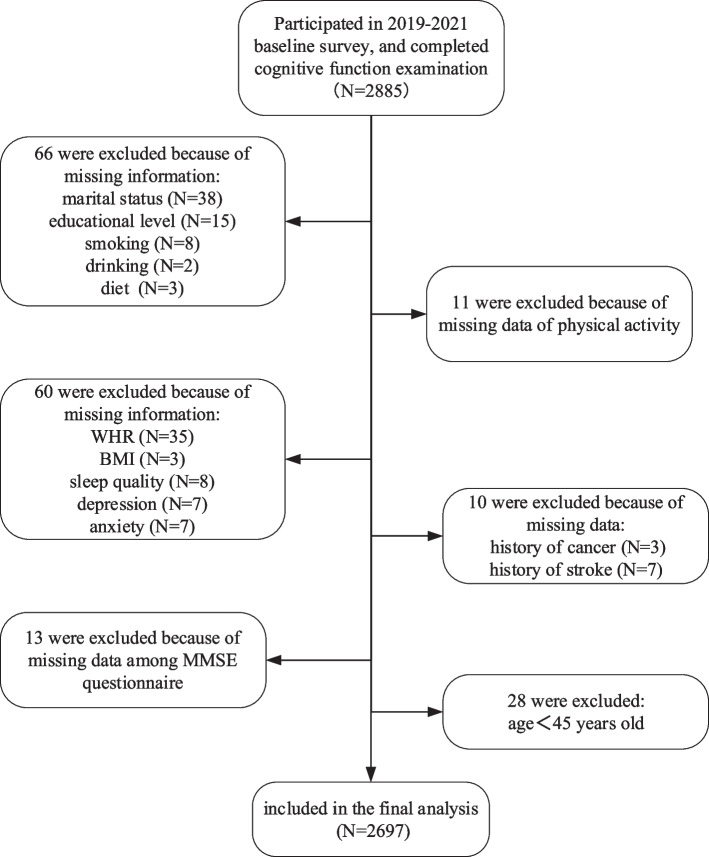
Study flow chart

Of the 2885 participants enrolled in this study, 188 (6.52%) were excluded due to missing data (Fig. 1). Finally, a total of 2697 subjects were included in the analyses (mean age: 66.19 years; 61.48% female). The mean MMSE score was 27.23 ± 2.72, with a prevalence of MCI of 8.60% (232 participants). For LTPA status, approximately 76.79% of the participants took LTPA, and the main types of LTPA were walking (1340 subjects, 49.68%), square dancing (270 subjects, 10.01%), and walking + square dancing (172 subjects, 6.38%). Every week, they spent a median of 7.0 hours doing LTPA, resulting in a median amount of 24.50 MET-hours, of which 70.37% reached the activity level recommended by the WHO. Other characteristics were shown in Table 1. Those who suffered MCI seemed to perform less LTPA either in terms of total amount or duration (Supplementary Fig. 1).

**Table 1:** Characteristics of participants

Characteristics	(***N*** = 2697)
Age, mean(SD)	66.19 (6.68)
Sex, n(%)	
Male	1039 (38.52)
Female	1658 (61.48)
Location, n(%)	
Rural	1315 (48.76)
Urban	1382 (51.24)
Education level, n(%)	
Primary school and below	1224 (45.38)
Middle school	852 (31.59)
High school or vocational high school	482 (17.87)
College and above	139 (5.15)
Marital status, n(%)	
Unmarried/divorced	363 (13.46)
Married/cohabitation	2334 (86.54)
Smoke, n(%)	
Never	2155 (79.90)
Former	176 (6.53)
Current	366 (13.57)
Drink, n(%)	
Never	1902 (70.52)
Former	128 (4.75)
Current but not heavy drinker	627 (23.25)
Heavy drinker	40 (1.48)
Diet, n(%)	
Bland	1428 (52.95)
Moderate	750 (27.81)
Spicy	206 (7.64)
Salty	92 (3.41)
Others	221 (8.19)
Central obesity, n(%)	
No	827 (30.66)
Yes	1870 (69.33)
Overweight, n(%)	
No	1078 (39.97)
Yes	1619 (60.03)
Total amount of LTPA (MET-hours/week), n(%)	
< 11.25	799 (29.63)
11.25 ~ 24.50	377 (13.98)
24.50 ~ 36.40	840 (31.14)
≥ 36.40	681 (25.25)
Duration of LTPA (h/week), n(%)	
< 2.5 h	799 (29.63)
2.5 ~ 7.0 h	397 (14.72)
7.0 ~ 10.5 h	1007 (37.34)
≥ 10.5 h	494 (18.32)
Type of LTPA, n(%)	
None	626 (23.21)
Walking	1340 (49.68)
Square dancing or other aerobic dancing	270 (10.01)
Walking plus dancing	172 (6.38)
Others	289 (10.72)
Sleep quality, n(%)	
Good	1208 (44.79)
Not bad	1006 (37.30)
General/poor	483 (17.91)
Depression, n(%)	
No	2558 (94.85)
Yes	139 (5.15)
Anxiety, n(%)	
No	2478 (91.88)
Yes	219 (8.12)
Hypertension, n(%)	
No	1201 (44.53)
Yes	1496 (55.47)
Diabetes, n(%)	
No	2256 (84.46)
Yes	415 (15.54)
Stroke, n(%)	
No	2581 (95.70)
Yes	116 (4.30)
Traumatic brain injuries, n(%)	
No	2648 (98.18)
Yes	49 (1.82)
Cancer, n(%)	
No	2645 (98.07)
Yes	52 (1.93)
MMSE, mean(SD)	27.23 (2.72)
MCI, n(%)	
No	2465 (91.40)
Yes	232 (8.60)

### Association between LTPA and cognitive function

Linear regressions showed significant positive associations between LTPA and cognitive functions among middle-aged and older people. Compared with people who did not reach the WHO recommended activity level, middle-aged and elderly people who regularly participated in LTPA had better cognitive function (*P* = 0.001). Cognitive function increased with the amount of LTPA from 11.25 MET-hours/week to 36.40 MET-hours/week (*β* 0.09 for 11.25 ~ 24.50 MET-hours/week, *β* 0.38 for 24.50 ~ 36.40 MET-hours/week) but plateaued with higher amounts (*β* 0.39 for ≥36.40 MET-hours/week). For the duration of LTPA, a similar result was presented. Cognitive function increased with the duration of LTPA from 2.5 to 10.5 hours/week (*β* 0.11 for 2.5 ~ 7.0 hours/week, *β* 0.37 for 7.0 ~ 10.5 hours/week) and then stabilized at a higher duration (*β* 0.39 for ≥10.5 hours/week). Even for those who only walked, a positive association between LTPA and cognitive function still existed, and at least 7.0 ~ 10.5 h/week or 24.50~36.40 MET-hours/week of LTPA is recommended (table 2). For specific dimensions of cognitive function, the impact of LTPA was mainly reflected in orientation and language dimensions in terms of both total amount and duration of activity (supplementary Table 2).

**Table 2:** The relationship between LTPA and cognitive function among middle-aged and older people in Southwest China

Variables	N (2697)	MMSE (focus on all types of LTPA)		N (1966)	MMSE (focus on walking)	
		***β***(95%CI)	***P***		***β***(95%CI)	***P***
Total amount of LTPA (MET-hours/week)*						
< 11.25	799	0.00		731	0.00	
11.25 ~ 24.50	377	0.09 (−0.23, 0.41)	0.588	257	0.05 (− 0.32, 0.41)	0.806
24.50 ~ 36.40	840	0.38 (0.12, 0.63)	**0.003**	664	0.37 (0.09, 0.64)	**0.008**
≥ 36.40	681	0.39 (0.13, 0.66)	**0.004**	314	0.20 (−0.16, 0.56)	0.274
*P* for trend		**0.001**			**0.042**	
Duration of LTPA (h/week)*						
< 2.5 h	799	0.00		727	0.00	
2.5 ~ 7.0 h	397	0.11 (−0.21, 0.43)	0.488	261	0.05 (− 0.31, 0.42)	0.779
7.0 ~ 10.5 h	1007	0.37 (0.13, 0.61)	**0.003**	664	0.37 (0.10, 0.64)	**0.008**
≥ 10.5 h	494	0.39 (0.10, 0.68)	**0.008**	314	0.20 (−0.16, 0.56)	0.270
*P* for trend		**0.001**			**0.041**	

*adjusted for age, sex, WHR, sleep quality, education level, and marital status

**Fig. 2:**
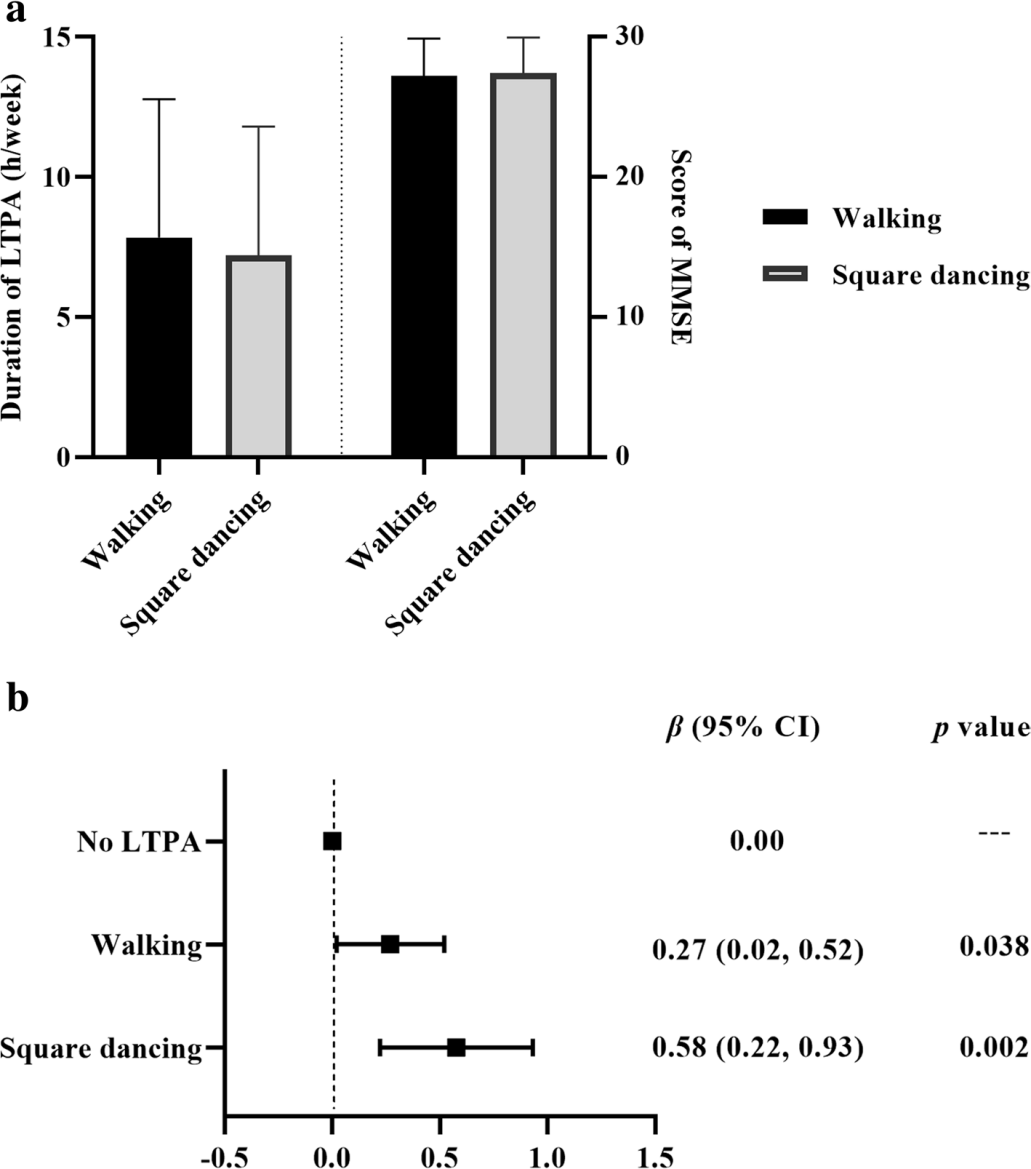
Characteristics of walking and square dancing of people aged 45 years or older in Southwest China and their impact on cognitive function. (**a**) Duration of LTPA (h/week) and score of MMSE for people who prefer walking or square dancing. (**b)**. Association between LTPA type (walking or square dancing) and cognitive function Multiple regression in Fig. 2b. was adjusted for age, WHR, sleep quality, education level, and marital status

As the two most popular LTPA for middle-aged and elderly people in Southwest China, those who prefer walking seemed to spend a longer leisure time than square dancing, with an average duration of 7.84 and 7.20 hours per week, respectively (Fig. 2a.). While the positive association with cognitive function appeared to be stronger in those who preferred square dancing than walking (*β* 0.27 for walking and *β* 0.58 for square dancing, Fig. 2b.).

### Subgroup analysis

**Fig. 3:**
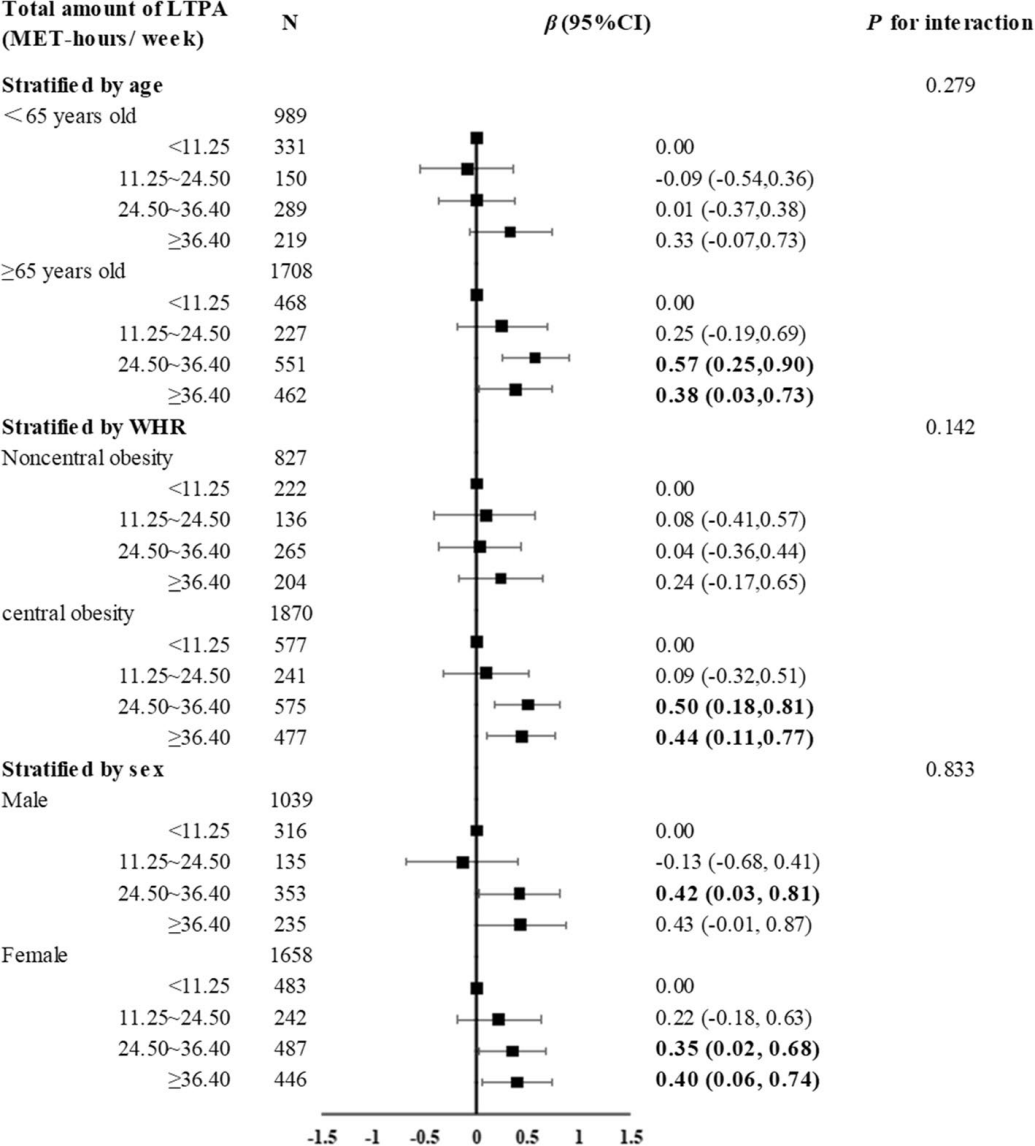
Subgroup analysis according to age, WHR and sex All models were adjusted for age, WHR, sleep quality, education level, and marital status

Although there was no statistically significant difference in the correlation of LTPA and cognition between each subgroup when we stratified by gender, age or WHR (*P* > 0.05), the association appeared to be stronger in people older than 65 years of age and with central obesity. In these two groups, the results also showed that with the increase in the LTPA amount, the correlation gradually increased and then tended to be stable or even slightly decreased (Fig. 3).

## Discussion

Our study is the first to evaluate the real-world LTPA pattern, cognitive function and their association among middle-aged and elderly people in Southwest China. We found that the MCI prevalence among people over 45 years old in this region is much lower than the domestic average, which may benefit from the high participation of these people in walking and square dancing-oriented LTPA.

This study showed that approximately 70.37% of middle-aged and elderly individuals in Southwest China met the activity standards recommended by the WHO, which was higher than the national average of 65.94% [24]. While the current prevalence rate of MCI in this region is 8.60%, which is lower than the average level of 15.5% in China [4]. Further analysis found that LTPA levels were positively correlated with cognitive function in these people. Compared with these adults who did not meet the recommended activity level, cognitive function increased with increasing levels of LTPA, but their association did not enhance with higher LTPA status (≥36.40 met-hours/week or ≥ 10.5 h). A pooled analysis of 10 cohort studies found a similar phenomenon; compared with older people who do not exercise regularly, performing 3.1 to 6.0 hours of physical activity or expending 9.1 to 18.0/MET-hours of energy per week may achieve the best protective effect on cognitive function, and a higher LTPA level was not equal to greater benefits [25]. One hypothesis is that the ability of older individuals to engage in physical activity gradually decreases due to age-related declines in the function of the cardiopulmonary and muscular systems. Additionally, the higher the dose of physical activity, the greater the risk of injury. Therefore, when recommending the optimal exercise dose for middle-aged and elderly people, the pros and cons should be weighed. More exercise may not always be better.

Moreover, we found that even for walking only, a positive correlation between LTPA and cognitive function was observed. One study from the CHARLS cohort showed that moderate-intensity physical activity (including brisk walking) was most beneficial to the successful aging of elderly individuals [26]. A meta-analysis suggested that the minimal clinically important amount of walking to improve cognitive function for the elderly was 13.27–14.18 met-hours/week [27]. Although compared with other forms of exercise, such as aerobic exercise and gyming, walking has a slightly weaker positive effect on neurological health [28], it is relatively safe and is currently the most popular exercise for the elderly in China [29]. Our results showed that square dancing was another popular exercise form among middle-aged and elderly people. This dance form is usually conducted in large public spaces, and consists of music, dance leader(s), and a group of dancers, which integrating Chinese style dancing and music with similar rhythms [12]. Most of our surveys began after the COVID-19 pandemic, so the number of people gathering for square dancing may have decreased. However, our preliminary findings suggested that square dancing, which was beneficial for the musculoskeletal system [30] and incorporated the pleasurable experience of social communication [31] and musical rhythm [32], may provide greater cognitive protection with less time spent exercising and thus could be regarded as a recommended method of LTPA for elderly individuals.

This study showed that the effects of LTPA on cognitive function were mainly reflected in orientation and language dimensions, both of which belong to executive function, while memory and recall ability belonged to memory function. Previous studies demonstrated that physical activity was associated with reduced brain atrophy [33], which may delay the decline in executive function and memory function in elderly individuals. However, the difference between the effect of exercise on executive and memory function is still controversial. A meta-analysis found that exercise had a stronger impact on memory function than executive function [34]. While the study of Baker et al. showed that the cognitive enhancement effect of aerobic exercise was limited to the executive control process, excluding declarative memory [35]. Differences in results may be due to the difference in study design, population selection, measurement methods of exposure and outcome, etc., which need further study.

Our further subgroup analysis found that the association between LTPA and cognitive function seemed to be more pronounced in those with central obesity and older adults. Similarly, a previous study in Shanghai, China, suggested that waist circumference control and appropriate physical activity can help improve the cognitive level of the elderly [36]. Since moderate physical activity is also beneficial for waist circumference control, the effect of exercise on cognitive function may be more pronounced in people with central obesity. In addition, the cognitive function of the elderly was generally worse than that of the middle-aged, and there was more room for improvement, so the positive effect of physical activity may be more obvious.

There are several limitations in this study. First, given the cross-sectional design, our study had a limited ability to demonstrate a causal association between LTPA and cognitive function. However, we investigated the LTPA status of the subjects during the past 6 months, which helped to avoid the risk of causal inversion to a certain extent. In addition, although only three cities in Sichuan Province were investigated, we took into account areas with different income levels to maximize the representativeness of the participants under limited resources. Despite the above limitations, this study used a relatively large sample size and demonstrated the positive impact of the LTPA mode based on walking and square dancing on cognitive function. Our results will be helpful for the formulation of behavioral intervention programs for dementia. In the future, we intend to improve the survey of sedentary conditions, commuting-related activities, etc., to more accurately describe the amount of PA of the population and perform a follow-up study in this region.

## Conclusions

The LTPA level of middle-aged and elderly people in Southwest China is generally high, with walking and square dancing being the major leisure physical activities. LTPA has a positive impact on cognitive function, with the effect mainly reflected in the language and orientation dimensions. At least 24.50 MET-hours/week of LTPA is recommended, while aimed at cognition improvement for middle-aged and elderly people.

## Supplementary Information

**Additional file 1: Supplementary Table 1.** Physical activity types, codes and MET values. **Supplementary Table 2.** The correlation between LTPA status and different dimensions of cognitive function. **Supplementary Figure 1a.** Characteristics of total amount and duration of LTPA among MCI and non-MCI participants. **Supplementary Figure 1b.** Association between total amount of LTPA and risk of MCI. **Supplementary Figure 1c.** Association between duration of LTPA and risk of MCI. **Supplementary Figure 2a.** Characteristics of MMSE score for participants with different total amount of LTPA. **Supplementary Figure 2b.** Mean score of MMSE for participants with different total amount of LTPA. **Supplementary Figure 2c.** Characteristics of MMSE score for participants with different duration of LTPA. **Supplementary Figure 2d.** Mean score of MMSE for participants with different duration of LTPA.

## Data Availability

The datasets of the current study are available from the corresponding author upon reasonable request.

## Abbreviations

LTPA: leisure-time physical activity
BMI: Body mass index
WHR: waist-to-hip ratio
MCI: mild cognitive impairment
METs: metabolic equivalents
MMSE: Mini-Mental State Examination.
