# Supplementary material for: Association between walking and square dancing-oriented leisure-time physical activity and cognitive function among middle-aged and elderly people in Southwest China
Source: BMC Geriatr. 2023 Jan 16;23:28. doi: 10.1186/s12877-023-03737-0 (PMC9841134; doi:10.1186/s12877-023-03737-0)
Supplement: Supplementary file 1 — Additional file 1: Supplementary Table 1. Physical activity types, codes and MET values. Supplementary Table 2. The correlation between LTPA status and different dimensions of cognitive function. Supplementary Figure 1a. Characteristics of total amount and duration of LTPA among MCI and non-MCI participants. Supplementary Figure 1b. Association between total amount of LTPA and risk of MCI. Supplementary Figure 1c. Association between duration of LTPA and risk of MCI. Supplementary Figure 2a. Characteristics of MMSE score for participants with different total amount of LTPA. Supplementary Figure 2b. Mean score of MMSE for participants with different total amount of LTPA. Supplementary Figure 2c. Characteristics of MMSE score for participants with different duration of LTPA. Supplementary Figure 2d. Mean score of MMSE for participants with different duration of LTPA. [file 12877_2023_3737_MOESM1_ESM.docx]

**Supplementary Table 1. Physical activity types, codes and MET values**

| **Activity type** | **MET** | **codes** |
| --- | --- | --- |
| Jogging | 7.5 | 12020; 12025 |
| Brisk walking | 4.3 | 17200 |
| walking | 3.5 | 17160 |
| Gymnasium Activities | 6.2 | 02019; 02020*; 02022*; 02024*; 02140; 03019; 02080; 02117; 02010^#^; 02011^#^; 02012^#^; 02013^#^; 02014^#^; 02015^#^; 02017^#^ |
| Yoga | 3.0 | 02150; 02160; 02170; 02180 |
| Swimming | 7.5 | 18230; 18240; 18255; 18265; 18270; 18280; 18290; 18300; 18310; 18320; 18330; 18340; 18350 |
| Square dancing | 5.2 | 03015; 03025; 15300 |
| Ball games | 6.1 | 15030; 15040^&^; 15050^&^; 15055^&^; 15610; 15660; 15675 |
| Bicycle | 5.9 | 01010; 01015; 01018; 01019; 01020; 01030 |
| Other exercise, e.g. fishing, mountain walking,and Tai-Chi | 4.3 | 02064; 04001; 15425; 15537; 15670 |

The MET value of each code was obtained from the 2011 Compendium of Physical Activities, and we calculated the average MET value for each category of activity.

^*^ / ^#^ / ^&^: Because these items belong to one type of activity, the average of the small category is calculated first, and then the overall average is calculated.

Abbreviations: MET, Metabolic equivalent of tasks.

**Supplementary Table 2. The correlation between LTPA status and different dimensions of cognitive function**

| **variables** | **N**  **(2697)** | **orientation** | |  | **registration** | |  | **Attention and calculation** | |  | **recall** | |  | **language** | |
| --- | --- | --- | --- | --- | --- | --- | --- | --- | --- | --- | --- | --- | --- | --- | --- |
|  |  | **β（95%CI）** | ***P*** |  | **β（95%CI）** | ***P*** |  | **β（95%CI）** | ***P*** |  | **β（95%CI）** | ***P*** |  | **β（95%CI）** | ***P*** |
| **Total amount of LTPA (MET-hours/week)*** | |  |  |  |  |  |  |  |  |  |  |  |  |  |  |
| <11.25 | 799 | 0.00 |  |  | 0.00 |  |  | 0.00 |  |  | 0.00 |  |  | 0.00 |  |
| 11.25~24.50 | 377 | 0.06(-0.04, 0.16) | 0.257 |  | -0.02(-0.06, 0.01) | 0.188 |  | 0.03(-0.10, 0.17) | 0.629 |  | 0.01(-0.08, 0.10) | 0.842 |  | 0.01(-0.14, 0.16) | 0.897 |
| 24.50~36.40 | 840 | 0.12(0.04, 0.20) | **0.003** |  | 0.01(-0.01, 0.04) | 0.262 |  | 0.01(-0.10, 0.12) | 0.839 |  | 0.09(0.02, 0.15) | **0.016** |  | 0.15(0.04, 0.27) | **0.009** |
| ≥36.40 | 681 | 0.16(0.08, 0.24) | **<0.001** |  | 0.01(-0.02, 0.03) | 0.671 |  | -0.02(-0.14, 0.10) | 0.734 |  | 0.05(-0.02, 0.13) | 0.148 |  | 0.19(0.07, 0.31) | **0.002** |
| *P* for trend |  | **<0.001** |  |  | 0.321 |  |  | 0.741 |  |  | **0.046** |  |  | **<0.001** |  |
| **Duration of LTPA (h/week)*** |  |  |  |  |  |  |  |  |  |  |  |  |  |  |  |
| <2.5 h | 799 | 0.00 |  |  | 0.00 |  |  | 0.00 |  |  | 0.00 |  |  | 0.00 |  |
| 2.5~7.0 h | 397 | 0.05(-0.05, 0.15) | 0.303 |  | -0.02(-0.05, 0.01) | 0.255 |  | 0.04(-0.09, 0.17) | 0.563 |  | 0.02(-0.07, 0.10) | 0.741 |  | 0.02(-0.12, 0.17) | 0.752 |
| 7.0~10.5 h | 1007 | 0.13(0.05, 0.21) | **<0.001** |  | 0.01(-0.01, 0.04) | 0.280 |  | -0.01(-0.11, 0.10) | 0.914 |  | 0.08(0.01, 0.14) | **0.027** |  | 0.16(0.05, 0.27) | **0.004** |
| ≥10.5 h | 494 | 0.15(0.06, 0.24) | **<0.001** |  | 0.004(-0.03, 0.04) | 0.801 |  | -0.002(-0.13, 0.13) | 0.979 |  | 0.06(-0.02, 0.14) | 0.125 |  | 0.18(0.05, 0.31) | **0.008** |
| *P* for trend |  | **<0.001** |  |  | 0.387 |  |  | 0.860 |  |  | **0.034** |  |  | **0.001** |  |

*adjusted for age, sex, WHR, sleep quality, education level, and marital status.

| 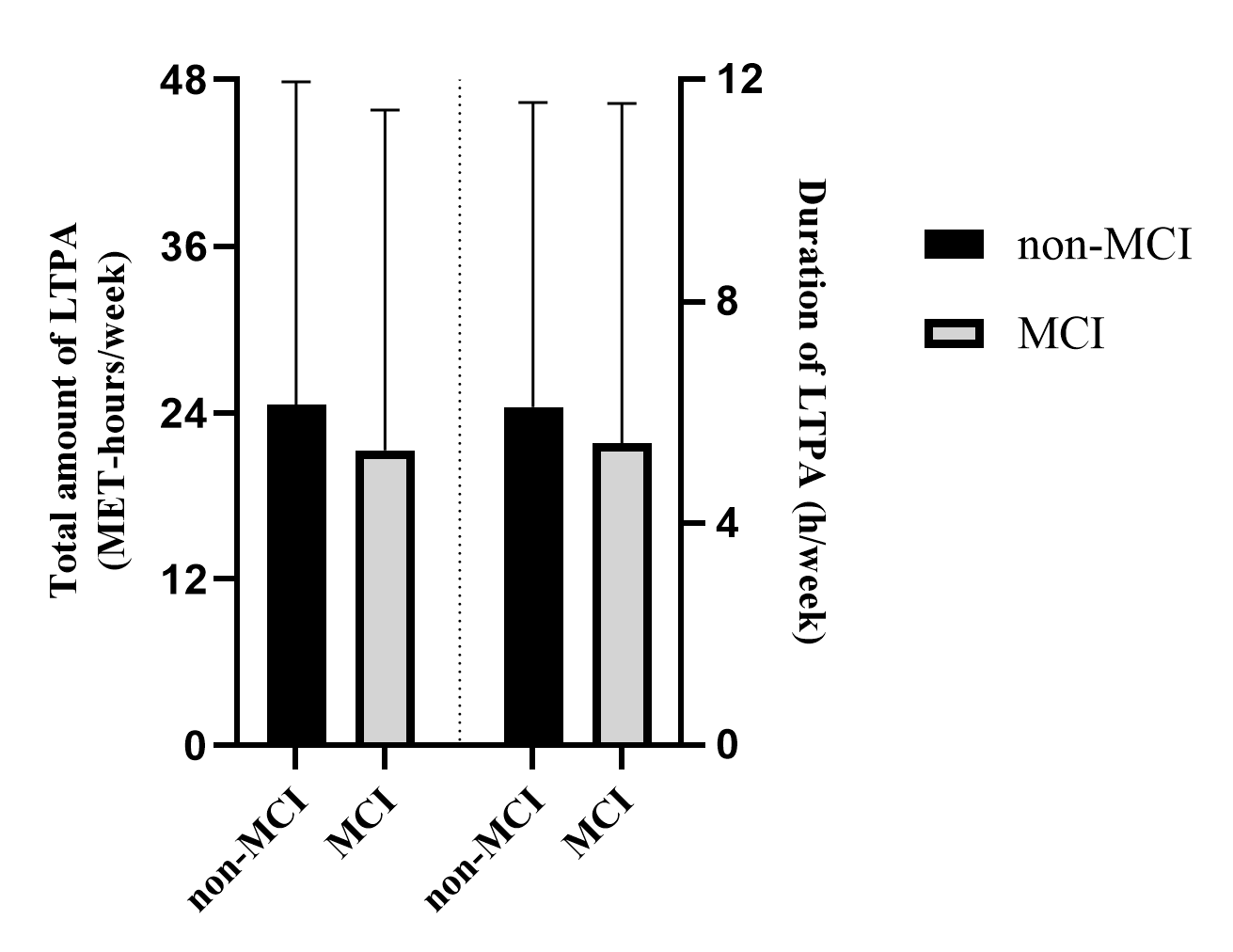 |
| --- |
| **Supplementary Figure 1a. Characteristics of total amount and duration of LTPA among MCI and non-MCI participants.** |

| 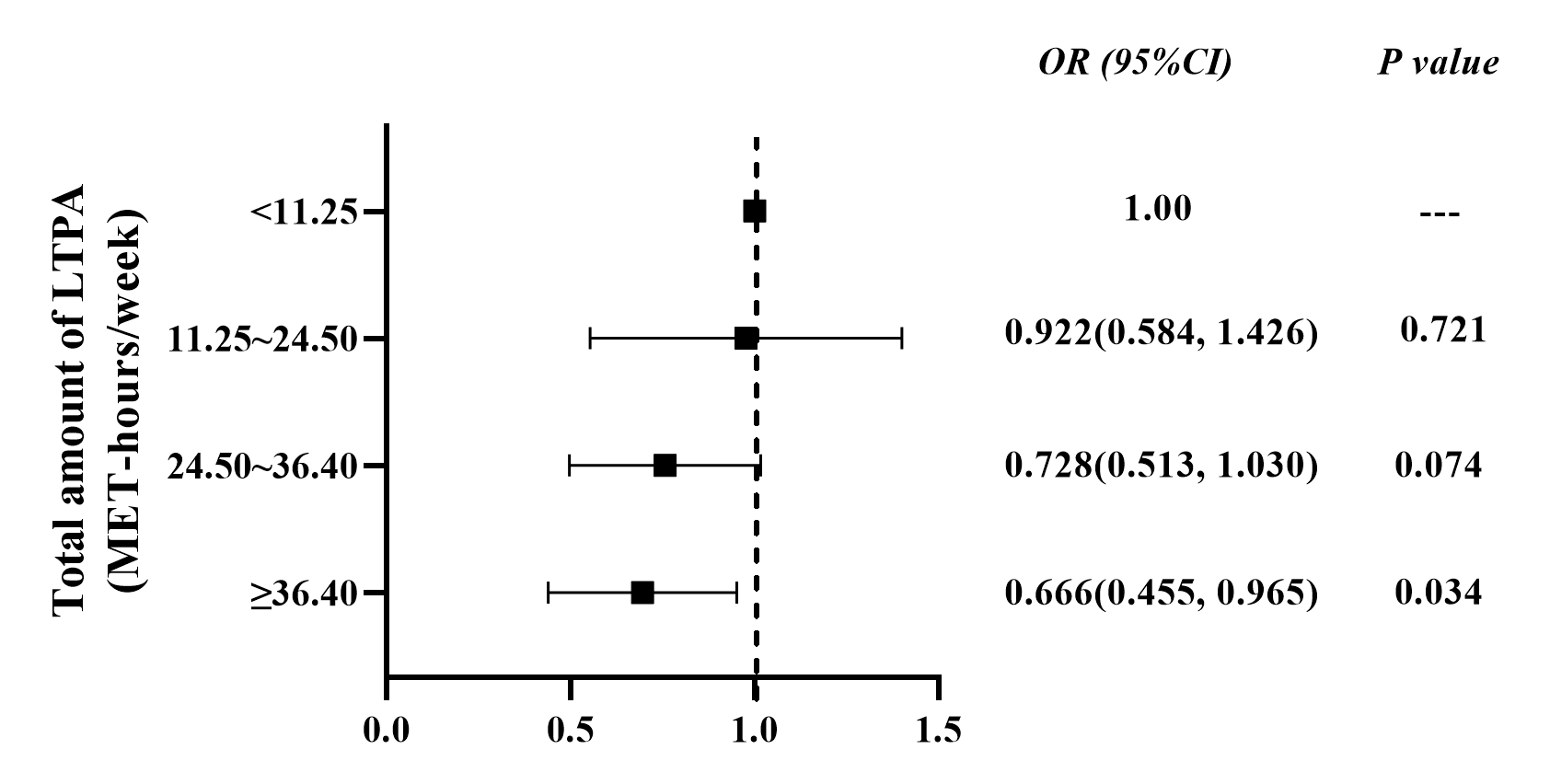 |
| --- |
| **Supplementary Figure 1b. Association between total amount of LTPA and risk of MCI** |
| Multiple logistic regression was adjusted for age, WHR, sleep quality, education level, and marital status. |

| 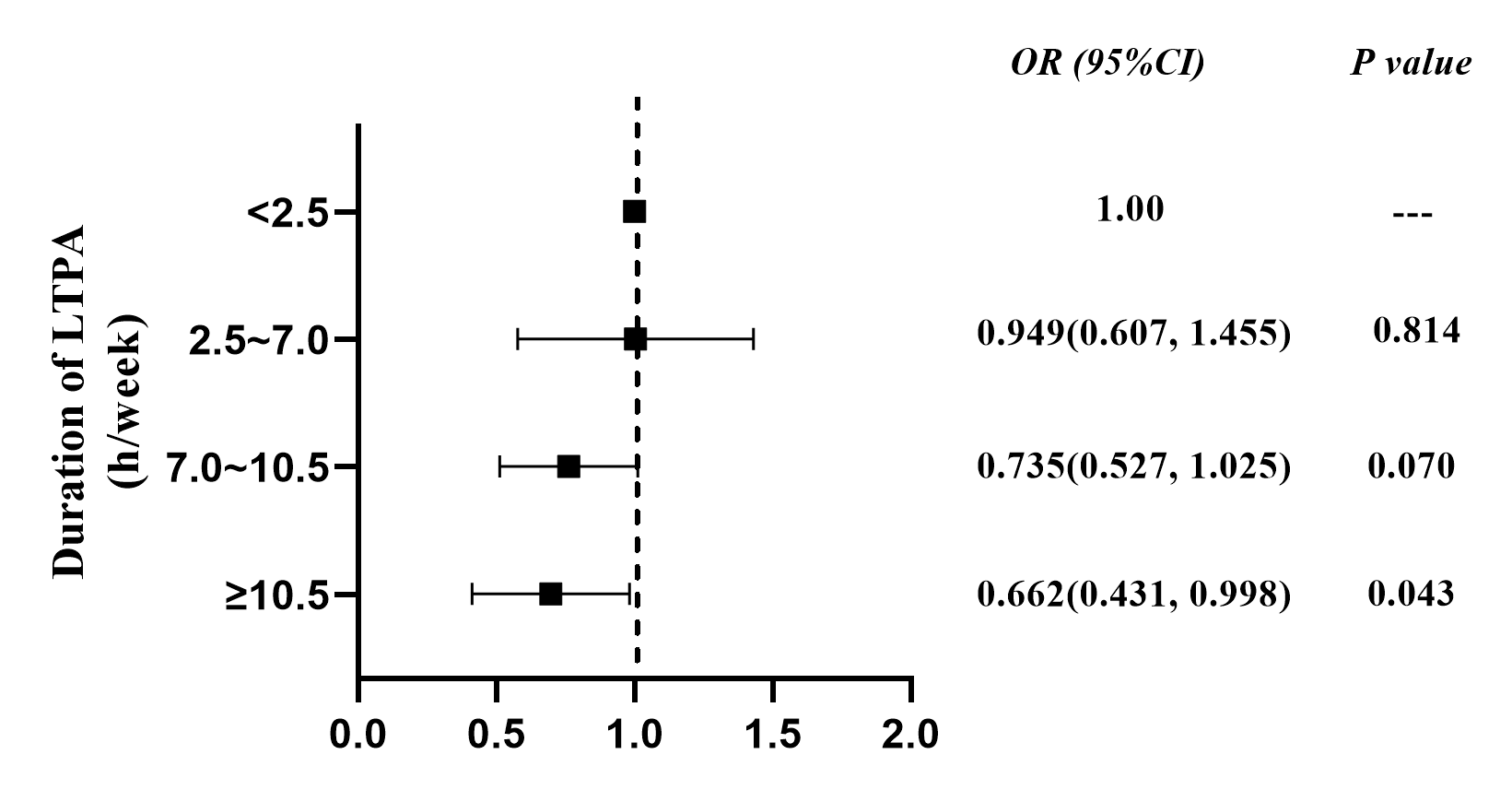 |
| --- |
| **Supplementary Figure 1c. Association between duration of LTPA and risk of MCI** |
| Multiple logistic regression was adjusted for age, WHR, sleep quality, education level, and marital status. |

| 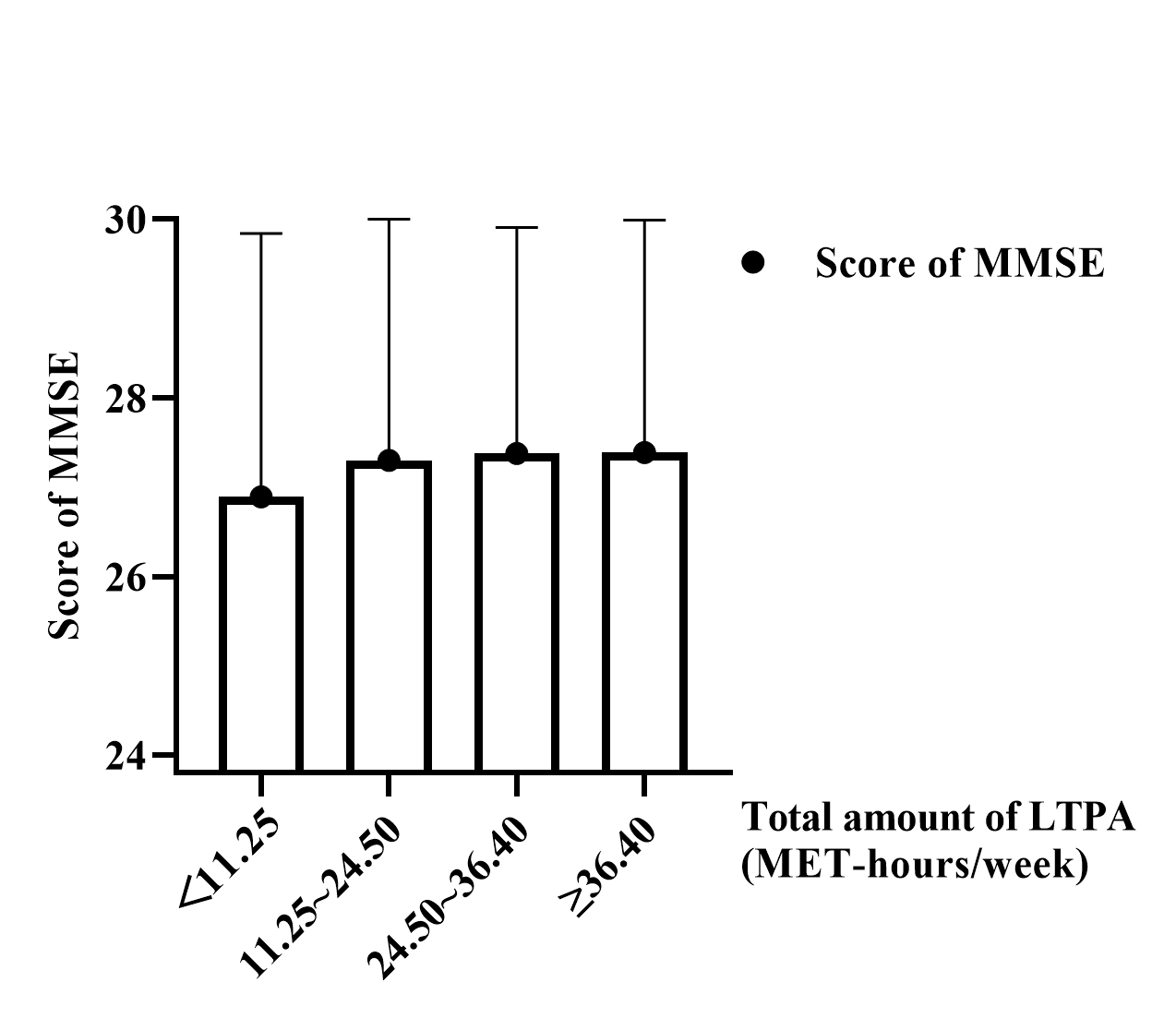 | 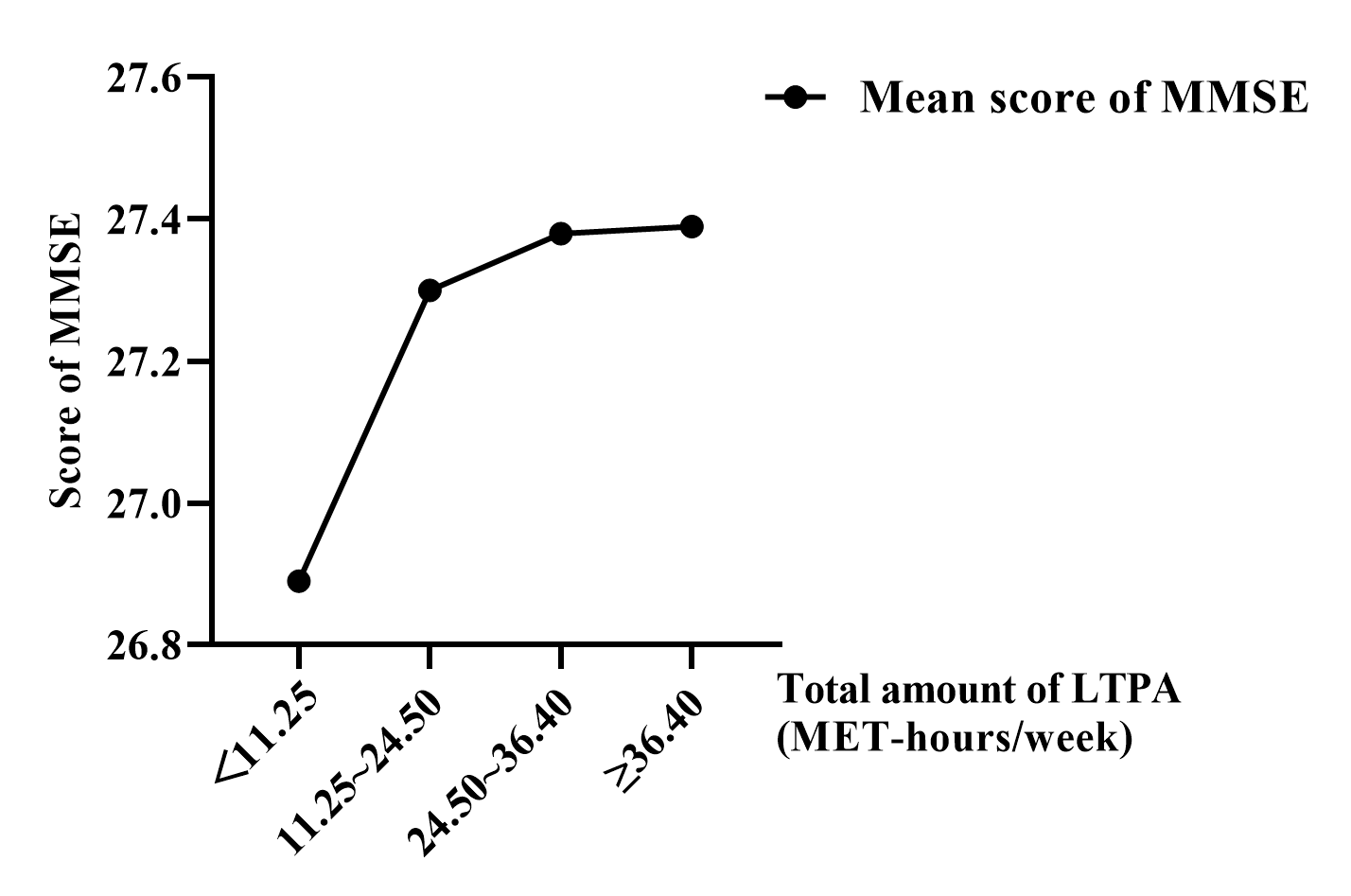 |
| --- | --- |
| **Supplementary Figure 2a.** Characteristics of MMSE score for participants with different total amount of LTPA | **Supplementary Figure 2b**. Mean score of MMSE for participants with different total amount of LTPA |

| 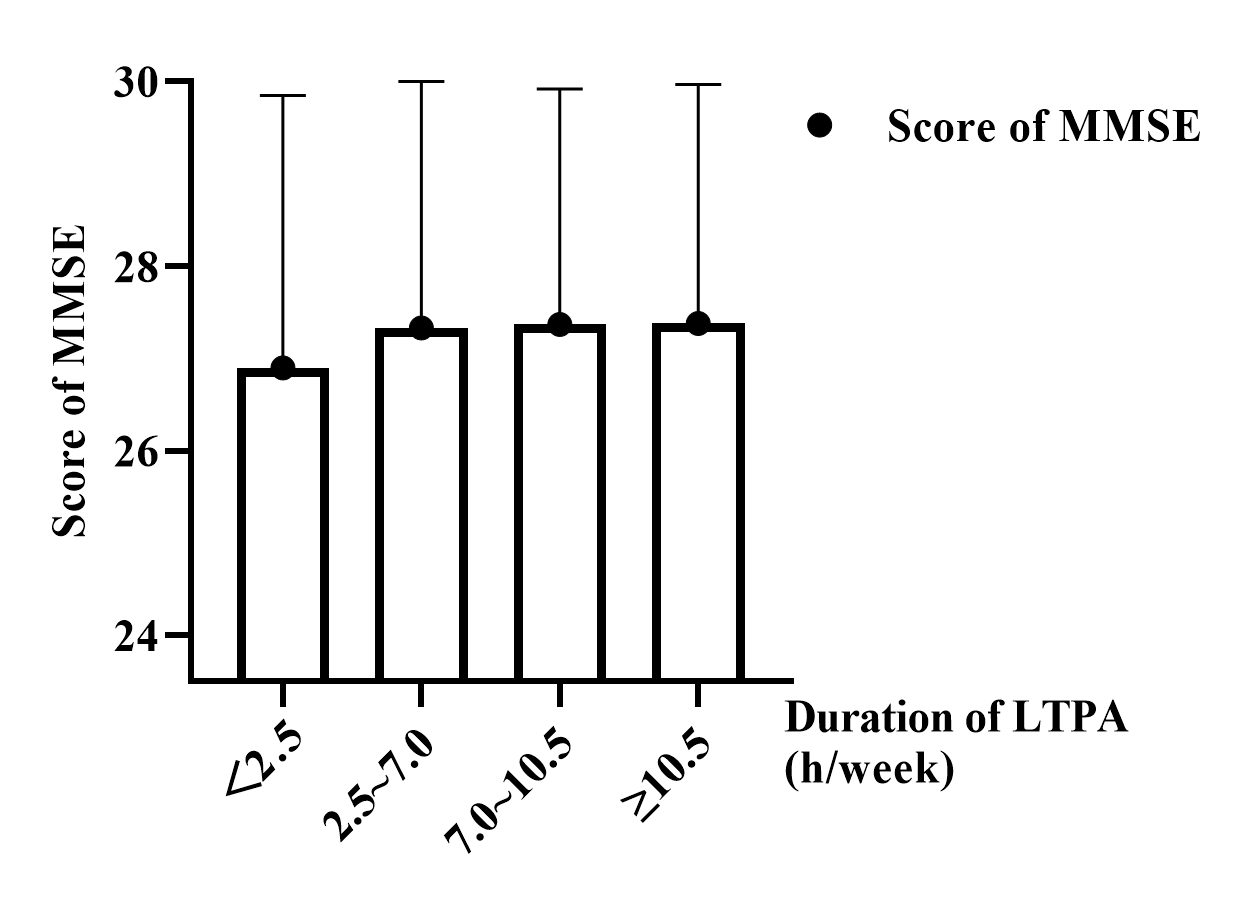 | 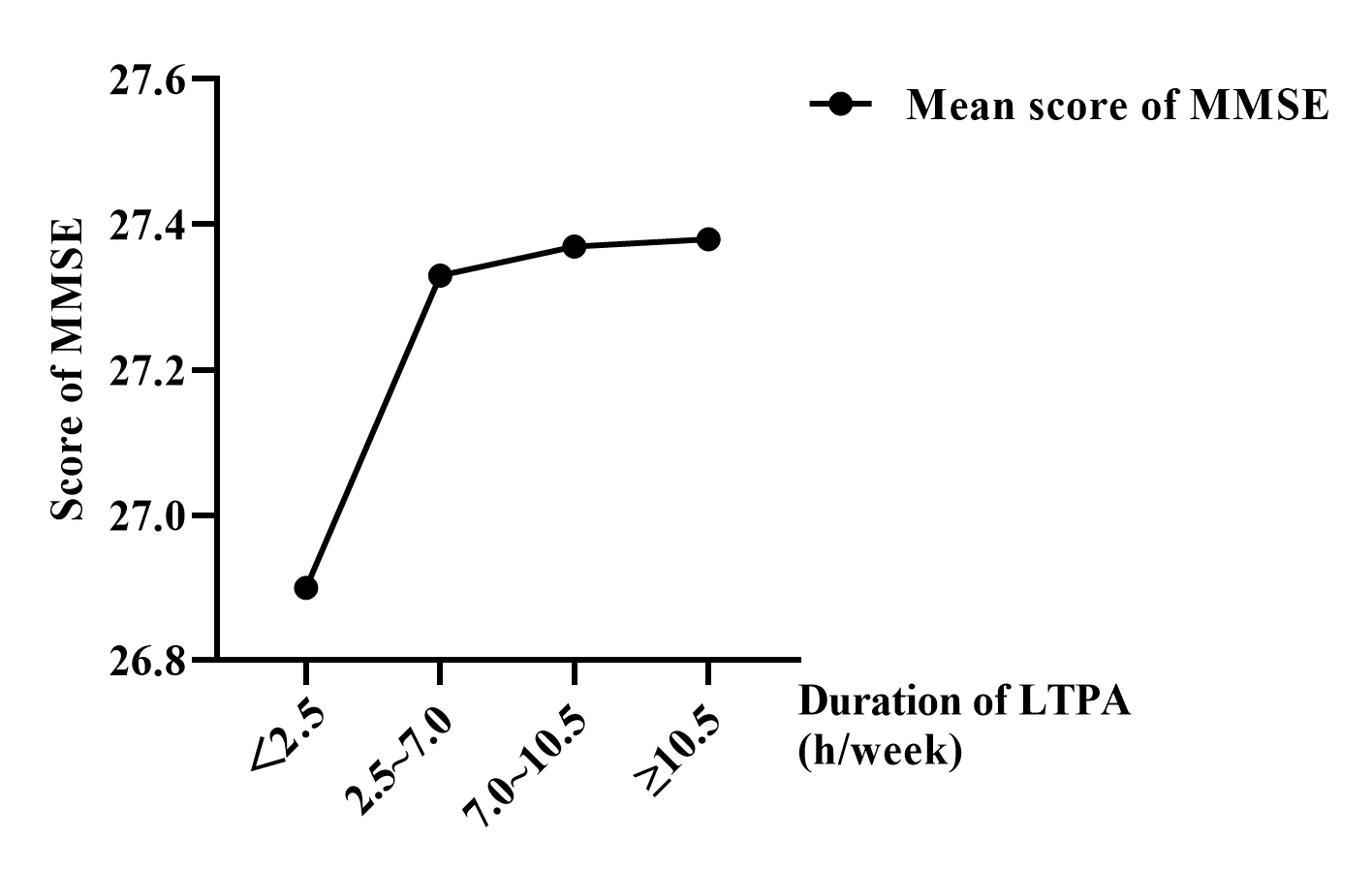 |
| --- | --- |
| **Supplementary Figure 2c.** Characteristics of MMSE score for participants with different duration of LTPA | **Supplementary Figure 2d**. Mean score of MMSE for participants with different duration of LTPA |
